# Evolution of single gyroid photonic crystals in bird feathers

**DOI:** 10.1073/pnas.2101357118

**Published:** 2021-05-31

**Authors:** Vinodkumar Saranathan, Suresh Narayanan, Alec Sandy, Eric R. Dufresne, Richard O. Prum

**Affiliations:** ^a^Division of Science, Yale-NUS College, National University of Singapore, 138609 Singapore;; ^b^Department of Ecology and Evolutionary Biology, Yale University, New Haven, CT 06520;; ^c^Peabody Museum of Natural History, Yale University, New Haven, CT 06520;; ^d^Advanced Photon Source, Argonne National Laboratory, Lemont, IL 60439;; ^e^Department of Materials, ETH Zürich, 8093 Zürich, Switzerland

**Keywords:** biophotonic nanostructure, self-assembly, single gyroid, phase separation, bird coloration

## Abstract

Vivid, saturated structural colors are conspicuous and important features of many animals. A rich diversity of three-dimensional periodic photonic nanostructures is found in the chitinaceous exoskeletons of invertebrates. Three-dimensional photonic nanostructures have been described in bird feathers, but they are typically quasi-ordered. Here, we report bicontinuous single gyroid β-keratin and air photonic crystal networks in the feather barbs of blue-winged leafbirds (*Chloropsis cochinchinensis sensu lato*), which have evolved from ancestral quasi-ordered channel-type nanostructures. Self-assembled avian photonic crystals may serve as inspiration for multifunctional applications, as they suggest efficient, alternative routes to single gyroid synthesis at optical length scales, which has been experimentally elusive.

Many animals produce vivid, saturated structural colors via constructive light interference from diverse integumentary nanostructures with mesoscopic (∼100 nm to 350 nm) long-range order or short-range translational order (i.e., quasi-order) (1, 2). Structural coloration is an important aspect of their appearance, and often functions in social and sexual signaling (3). Physicists and engineers are increasingly interested in animal structural coloration as a source of inspiration for mesoscale manufacture (4, 5). A remarkable diversity of structural color-producing three-dimensional (3D) biophotonic nanostructures has been characterized within the chitinaceous exoskeletons of invertebrates (1, 2, 6). While vertebrates possess 1D and 2D periodic photonic nanostructures (1, 2), reported 3D photonic nanostructures are typically quasi-ordered, and limited to two classes of nanostructures that produce noniridescent or isotropic structural colors in feather barbs (7).

Here, we report single gyroid photonic crystal networks of β-keratin and air within brilliantly colorful blue and green feather barbs of the Blue-winged Leafbird (*Chloropsis cochinchinensis s. l.*, Chloropseidae), revealed by synchrotron small-angle X-ray scattering (SAXS) and scanning electron microscopy (SEM). We compare these ordered morphologies to homologous channel-type nanostructures with short-range order and intermediate structures from other closely related *Chloropsis* species, and their sister group, the fairy bluebirds (*Irena,* Irenidae) (7, 8).

## Results

**Fig. 1.**
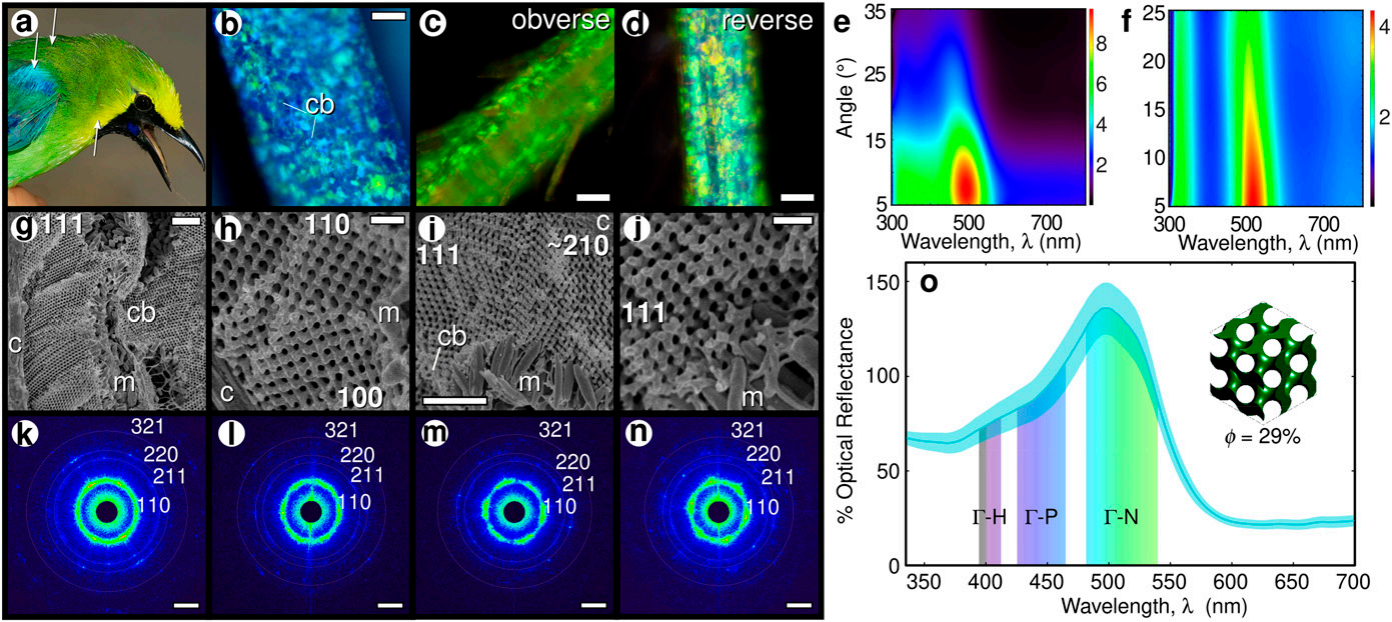
Single gyroid photonic crystals in the plumage of Blue-winged Leafbird (*C. cochinchinensis kinneari*). (*A*) Photograph with approximate sampling locations (white arrows). Image credit: John C. Mittermeier (photographer). Representative light micrographs with iridescent highlights (*B*−*D*) and angle-resolved specular reflectance measurements (false color) (*E* and *F*), SEM images (*G*−*J*), and SAXS diffraction patterns (*K*−*N*) of brilliant blue epaulet (*B*, *E*, *G*, *H*, *K*, and *L*) and green back (*C*, *D*, *F*, *I*, *J*, *M*, and *N*) feather barbs. Crystallite domain orientations are indicated in SEM images (*G*–*J*); c, cortex; cb, cell boundary; m, melanosomes. SAXS patterns (*K*–*N*) are depicted in a log-scale, false-color encoding and indexed as a single network gyroid (*I*4_1_32) by the diagnostic presence of 110, 211, 220, and 321 peaks (white concentric circles) and key absence of the 200 peak per standard International Union of Crystallography conventions (6, 9) (Fig. 2*A*). (*O*) Microspectrophotometric measurements (turquoise line: mean with std. error envelope) of blue epaulet barbs are congruent with photonic bandgap modeling (shaded pseudogaps) of a single gyroid (*Inset*). (Scale bars: *B*–*D*, 20 μm; *G* and *I*, 2 μm; *H* and *J*, 500 nm; *K*–*N*, 0.025 nm^−1^.)

**Fig. 2.**
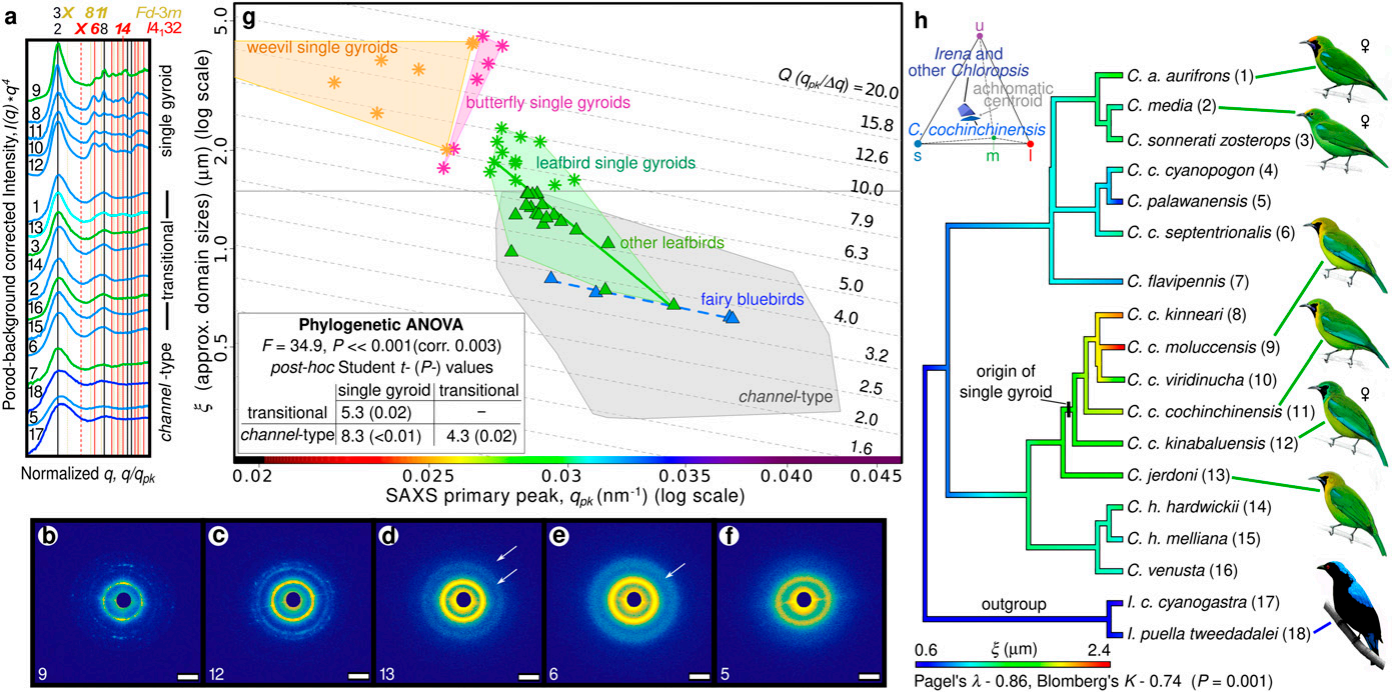
Evolutionary disorder-to-order transition in leafbird feathers. (*A*) Porod background-corrected, normalized, azimuthally averaged SAXS profiles and (*B*–*F*) a representative subset of the corresponding diffraction patterns of homologous feather barbs from all *Chloropsis* and two *Irena* species (8), in order of decreasing long-range translational order (*ξ*). Profile colors reflect the approximate hue of assayed plumage patches, and numbers correspond to taxa labels in *H* (Dataset S1). (Scale bars, 0.025 nm^−1^.) White arrows in *D* and *E* indicate higher-order features seen in transitional barb nanostructures. Labeled vertical lines in *A* (X/dashed line: forbidden; bold italics/colored line: diagnostic) denote expected Bragg reflections for alternative cubic space groups (6, 9). (*G*) Ashby diagram with nanostructural *Q* factors (*q*_*pk*_*/*Δ*q*, a measure of spectral purity) plotted as scale-independent (dashed gray) isolines in equally spaced decidecades. Plotted alongside leafbird single gyroids (*) and transitional and ancestral channel-type nanostructures of other leafbirds (green ▲) and fairy bluebirds (blue ▲) are the gamut (shaded convex hulls) of known avian channel*-*type nanostructures (7), and self-assembled visible length-scale single gyroids in butterflies (9) and weevils (6). Linear regressions of log-transformed data for fairy bluebirds (dashed blue line) and leafbirds (green line) show within-genera trajectories. The spectrum on the *x* axis is an approximate color guide to barb hues, for a given *q*_*pk*_. *Inset* shows the results of phylogenetic ANOVA. (*H*) Bayesian ancestral state reconstruction of *ξ* on a consensus molecular phylogeny of *Chloropsis* and *Irena* (8), with a single origin of single gyroid marked in the Blue-winged Leafbird clade (*C. cochinchinensis s. l.*). (*Inset*) Avian visual modeling shows that blue epaulet colors of *C. cochinchinensis*, on average, have lower ultraviolet signal and higher chroma than comparable blue patches of other leafbirds and *Irena* (Dataset S1). Illustrations reprinted from ref. 16, which is licensed under CC BY 4.0.

Light micrographs of Blue-winged Leafbird feathers show iridescent highlights within individual barb cells (Fig. 1 *B*–*D*). Angle-resolved spectral measurements document weak iridescence (Fig. 1 *E* and *F*). SEM images (Fig. 1 *G*–*J*) reveal ordered interconnected mesoporous networks of β-keratin rods and air channels with a polycrystalline texture, underlain by a dense layer of basal melanosomes to absorb any unscattered light. The SAXS diffraction patterns generally exhibit sixfold symmetries and up to eight orders of discrete Bragg spots (Figs. 1 *K*–*N* and 2 *A*–*C*) diagnosable as a single gyroid (*I*4_1_32) space group (6, 9). The observed microspectral reflection peak of a blue epaulet feather is consistent with the expected photonic bandgap structure of an appropriately sized single gyroid (9) (Fig. 1*O*).

Coherence length (*ξ* ≈ 2*π/∆q*, where Δ*q* is full width at half maximum [FWHM] of structural correlation peaks) is a measure of crystallite domain size, and thus the extent of long-range translational order (6, 9). To investigate the evolution of single gyroids in *C. cochinchinensis*, we plot the coherence lengths of barb nanostructures from all *Chloropsis* and *Irena* species (8), in addition to known diversity of other avian channel-type nanostructures (7), against their peak structural correlations (*q*_*pk*_) (Fig. 2*G*). Interestingly, the diversity of barb nanostructures among *Chloropsis* species (green line) exhibits a continuum of intermediate states (green triangles), from ancestral channel-type nanostructures of *Irena* (blue dashed line) to the derived single gyroids of *C. cochinchinensis* (green asterisks, Fig. 2*G*). The quasi-ordered channel-type nanostructures within *Irena* and the two sexually monomorphic species of *Chloropsis* (*palawanensis* and *flavipennis*) (8) exhibit nearly constant peak widths (FWHM, *Δq*) relative to their dominant length scales (*q*_*pk*_), *Q = q*_*pk*_*/*Δ*q* ≈ 4, suggesting that scale-invariant or size-independent processes (10) underlie the assembly of quasi-ordered nanostructural states in these species. The evolution of nanostructural order from quasi-disorder within *Chloropsis* is apparent as a significant deviation from the scale-independent trend in Fig. 2*G*, characterized by a sharpening of the structural correlation peaks (Fig. 2 *A*–*F*).

Because the plumage photonic nanostructures of *Chloropsis* and *Irena* are homologs that first evolved in the most recent common ancestor of these two monophyletic sister genera (7, 8), our comparative analysis demonstrates that the single gyroid photonic networks of blue-winged leafbirds were evolutionarily derived from ancestral quasi-ordered channel-type networks, still present in *Irena* (Fig. 2*H*). Moreover, the variation in nanostructures among *Chloropsis* species indicates the evolutionary transition from disorder to order proceeds as a gradual appearance and sharpening of higher-order Bragg reflections (cf. refs. 6 and 9). In addition to a second-order feature that is typical of channel-type nanostructures (7), the azimuthal SAXS profiles of the transitional *Chloropsis* nanostructures exhibit one to two additional higher-order peaks at ratios close to √6 to √8, √14, and √22 to √24, but not √4 (Fig. 2*A*).

## Discussion

The origin of 3D photonic crystals from quasi-ordered nanostructures in blue-winged leafbirds is evolutionarily parallel with the derivation of 2D hexagonal columnar crystals in *Philepitta* from ancestral 2D quasi-ordered arrays of parallel collagen fibers in structurally colored skin of the most recent common ancestor with *Neodrepanis* (Philepittidae) (11). In both cases, evolutionary transitions from quasi-ordered to ordered photonic crystals with much narrower structural correlation peaks (Fig. 2 *A*–*G*, and figures 6 and 7 of ref. 11) result in the production of more saturated or purer hues that are readily perceivable by avian visual systems (3). These results suggest social or sexual selection on perceivable optical properties of biophotonic nanostructures (e.g., preferences for purer/more saturated hues) have likely driven evolutionary transitions in nanostructural spatial organization, resulting in the evolution of extraordinarily brilliant structural coloration (11).

Single gyroids that can exhibit large complete bandgaps have long been a target for photonic and photovoltaic engineering (reviewed in ref. 5), but synthetic self-assembly of single gyroids at visible length scales has proven elusive. In insect wing scales, single gyroid photonic crystals are hypothesized to be templated by the cooption of the innate ability of cell membranes to invaginate into core−shell double gyroid precursor networks (6, 9, 12). The chitinous cuticle polymerizes within extracellular space enclosed by the plasma membrane, leaving behind a single gyroid network of chitin in air, upon apoptosis. Current synthetic approaches to self-assemble single gyroids follow a similar symmetry-breaking pathway (reviewed in ref. 5), starting with a double gyroid or an alternating gyroid in a diblock or triblock copolymer and subsequent selective etching of the matrix and one of the two network phases. Synthetic single gyroids produced this way are limited to small lattice parameters (typically <100 nm). Synthetic lipid water systems also show cubic phases including double gyroid but with lattice parameters limited to just tens of nanometers (13).

Quasi-ordered channel-type photonic nanostructures of avian feather barbs are understood to self-assemble within medullary cells likely via self-arrested phase separation of polymerizing β-keratin from the cytoplasm, in the absence of any cytoskeletal prepatterns or membraneous precursor templates (reviewed in ref. 14). Our findings suggest that single gyroid photonic crystals may be efficiently synthesized at optical length scales through processes akin to bottom-up phase separation. Simulations (15) suggest that the phase behavior of patchy particles with mutual short-range attraction and long-range repulsion can include a metastable single gyroid phase. Future research into in vivo and in vitro arrested phase separation of colloidal solutions of charged proteins or similar polymers may provide novel insights into synthetic self-assembly of ordered mesoporous phases, including the single gyroid (4).

## Materials and Methods

Plumage sampling information is provided in Dataset S1. SAXS assays, SEM, spectrophotometry, and photonic bandgap modeling of biophotonic nanostructures have been described previously (6, 7, 9). Avian tetrachromat visual modeling of plumage colors and phylogenetic analyses of nanostructural evolution were performed in *R*. Further details are provided in *SI Appendix*, *SI Text*.

## Supplementary Material

Supplementary File

Supplementary File

## Data Availability

All study data are included in the article and Dataset S1.
