# Supplementary material for: Evolution of single gyroid photonic crystals in bird feathers
Source: Proc Natl Acad Sci U S A. 2021 May 31;118(23):e2101357118. doi: 10.1073/pnas.2101357118 (PMC8201850; doi:10.1073/pnas.2101357118)
Supplement: Supplementary File [file pnas.2101357118.sapp.pdf]

Supplementary Information for:

Evolution of Single Gyroid Photonic Crystals in Bird Feathers

Vinodkumar Saranathan[1,4]*, Suresh Narayanan[2], Alec Sandy[2], Eric R. Dufresne[3], and Richard O. Prum[4]

Affiliations:

[1]Division of Science, Yale-NUS College, National University of Singapore, 138609, Singapore.

[2]Advanced Photon Source, Argonne National Laboratory, Lemont, Illinois, 60439, USA.

[3]Department of Materials Science, ETH, Zurich, Switzerland

[4]Department of Ecology and Evolutionary Biology, and Peabody Museum of Natural History, Yale University, New Haven, CT 06520, USA.

*__Email__: Vinodkumar.Saranathan@aya.yale.edu

This file includes:

- Supplementary text
- Supplementary Dataset 1
- References for SI

Extended Methods

Specimens: The nanostructure and structural color production in feather barbs of all 10-15 putative *Chloropsis* (Chloropseidae) and 2 *Irena* (Irenidae) species(1) were analyzed in this study (see Dataset S1).

Synchrotron Small Angle X-ray Scattering (SAXS): In order to average over as few medullary barb cells as possible, pinhole SAXS experiments at either 10x10 or 15x15 μm (horizontal x vertical) beam size were performed in transmission geometry on feather barbs at beamline 8-ID-I of the Advanced Photon Source (Argonne National Labs) and acquired data was processed as per standard protocol(2-4).

Ashby diagram: In order to structurally compare across the diversity of barb photonic nanostructures, the coherence length ($\xi = 2\pi/\Delta q$) of each nanostructure is plotted against the corresponding structural correlation peak (q_{pk}) on a log-log scale. In this Ashby diagram(5), the selection criterion, namely structural Q-factors ($Q = q_{pk}/\Delta q$, a measure of spectral purity) are plotted as scale-independent isolines. For a given range of q values (abscissa; 0.02–0.05 nm^{-1}), the scale-independent coherence lengths (ordinate) are obtained by scaling each q_{pk} by the same constant fractional multiplier, c, *i.e.*, $\xi = 2\pi/(c*q_{pk})$. A series of such isolines of constant Q (= $1/c$) can be generated and plotted at equally-spaced intervals of deci-decades in log scale for a range of Q from 1 to 20 (corresponding to a range of c from 1 to 0.05) by using the following equation:

$$c = 1 / (e^x),$$

where x ranges from $log_e(1)$ to $log_e(20)$, *i.e.* 0 to ~3 in increments of a deci-decade ($log_e(10)/10$).

Scanning Electron Microscopy (SEM): For scanning electron microscopy (SEM), fractured barb samples were gold- or platinum-coated and imaged on a Hitachi SU-70 environmental SEM.

Angle-resolved spectrophotometry: Angle-resolved UV-VIS-NIR reflectance spectra (2 s integration time, 5x averaging) from individual feathers were measured in specular (θ-2θ) geometry(6) using a custom goniometer setup coupled to an Ocean Optics USB2000+ spectrophotometer and an Ocean Optics DH-BAL 2000 light source, as described elsewhere(3). Reflectance was calibrated using an Ocean Optics Spectralon matte white standard and a piece of matt-black velvet cloth as dark reference. The spectra were plotted as false-color heatmaps in Matlab (R2018b, Mathworks, Natick, MA).

Normal incidence spectrophotometry: UV-VIS-NIR normal incidence reflectance measurements (0.1 – 0.4 s integration time) were collected using an Ocean Optics USB2000+ spectrophotometer from a ~5 mm^2 illuminated plumage patch with a bifurcated probe and holder either from individual feathers (3x averaging and with a pulsed Xenon PX-2 lamp) or study skins (2-3 different locations per plumage patch and with an Ocean Optics DH-BAL 2000 light source), and analyzed as per standard protocol(3).

UV-VIS-NIR Microspectropectrophotometry and Light Microscopy: Normal-incidence UV-VIS-NIR reflectance microspectra of leafbird feather barbs were acquired using a uSight-2000-Ni microspectrophotometer (Technospex Pte. Ltd., Singapore). Spectra with usable range between 335-950 nm were collected using a high NA 100x objective from a ~1.5 μm sized spot (100 ms integration time, 40x averaging) and calibrated using an Avantes WS-2 matt-white standard. Light microscope images were recorded using the same microspectrometer setup at various magnifications, using a Touptek U3CMOS-05 camera.

Photonic Bandgap modeling: The MIT photonic bandgap package (MPB)(7) was used to calculate the first eight bands of a single gyroid photonic crystal, using a mesh-size of 5 and a resolution of 32 to discretize the unit cell(2). As in (2), the bandgap calculations were optimized to find the dielectric (β-keratin, refractive index = 1.58) volume fractions needed ($\phi = 0.29$) to produce a mid-gap frequency (0.6043) of the Γ-N (110) pseudgogap, as given by a / λ_{pk}, where a is the lattice parameter measured using SAXS and λ_{pk} is the microspectral peak hue of the blue epaulet of *C. cochinchinensis kinneari* (see Fig. 1o, and Dataset S1).

Avian tetrachromat visual modeling: In order to understand how leafbirds perceive hues produced by single gyroid and *channel*-type barb nanostructures, avian tetrachromat visual modeling(8) of the reflectance spectra was performed in *R* (version 3.6) using the *Pavo* package (version 2.4.0)(9). The relative stimulation (quantal catches) of the four photoreceptors (*u*, *s*, *m* and *l*) and the corresponding colorimetric parameters in avian tetrachromat color space (RGB hue or azimuth θ; UV hue or elevation ϕ; and chroma or saturation r, the magnitude of the position vector given by the distance from the achromatic centroid of the tetrahedron)(8) were computed with Common Starling (Passeroidea: *Sturnus vulgaris*) spectral sensitivities and default settings (homogeneous illuminance) (see Dataset S1). Passeroid species, including leafbirds and fairy bluebirds, generally have UV- rather than Violet-sensitive SWS1 opsins(10, 11).

Phylogenetic analyses: Bayesian continuous ancestral character state reconstructions of coherence lengths (ξ) and avian tetrachromat visual model parameters (see Dataset S1) were performed in *R* using the *anc.Bayes()* routine of the *phytools* package (version 0.7-47)(12), with 1,000,000 generations (20% burn-in). For *Chloropsis spp.*, we used the corresponding coherence lengths for blue epaulet feathers where present and green back feathers, if not. The average coherence length of the two *Irena* species was set as the informative prior for the root/ ancestral node of *Irena* and *Chloropsis*. Using the *contMap()* function, the ancestral state reconstructions were mapped onto the topology, which was the 50% majority rule consensus molecular phylogeny of *Irena* and *Chloropsis* presented in Fig. 6 of (1). Polytomies (placement of *C. flavipennis*, and the "*C. moluccensis*" clade)(1) were randomly resolved and zero branch lengths were set to 1/100 of the total tree length. Phylogenetic signal (Blomberg's *K,* n = 10,000 simulations; and Pagel's λ) was computed using the *phylosig()* routine. Phylogenetically-corrected analyses of variance (n = 10,000 simulations) and *post-hoc* pairwise tests with Holm-Bonferroni correction for multiple comparisons were performed using the *phylANOVA()* routine of *phytools*(12).

Dataset S1

A spreadsheet detailing bird study-skin sampling information as well as the optical reflectance, avian tetrachromatic color space and nanostructural parameters for all plumage patches of leafbirds and fairy bluebirds assayed in this study. For details of other avian *channel*-type barb nanostructures plotted in Fig. 2, see Tables S1 and S2 of (3); for butterfly and weevil single gyroids, see Table S1 of (2) and Table S1 of (4) respectively.

Supplementary References

1. Moltesen M, Irestedt M, Fjeldsa J, Ericson PG, & Jonsson KA (2012) Molecular phylogeny of Chloropseidae and Irenidae - cryptic species and biogeography. *Mol Phylogenet Evol* 65(3):903-914.
2. Saranathan V*, et al.* (2010) Structure, function, and self-assembly of single network gyroid (I4(1)32) photonic crystals in butterfly wing scales. *P Natl Acad Sci Usa* 107(26):11676-11681.

3. Saranathan V*, et al.* (2012) Structure and Optical Function of Amorphous Photonic Nanostructures from Avian Feather Barbs: A Comparative Small Angle X-ray Scattering (SAXS) Analysis of 230 Bird Species. *Journal of The Royal Society Interface* 9:2563–2580.
4. Saranathan V*, et al.* (2015) Structural Diversity of Arthropod Biophotonic Nanostructures Spans Amphiphilic Phase-Space. *Nano Lett* 15(6):3735-3742.
5. ASHBY M, F. & CEBON D (1993) Materials selection in mechanical design. *J. Phys. IV France* 03(C7):C7-1-C7-9.
6. Noh H*, et al.* (2010) How Noniridescent Colors Are Generated by Quasi-ordered Structures of Bird Feathers. *Advanced Materials* 22(26-27):2871-2880.
7. Johnson S & Joannopoulos J (2001) Block-iterative frequency-domain methods for Maxwell's equations in a planewave basis. *Opt. Express* 8(3):173-190.
8. Stoddard MC & Prum RO (2008) Evolution of avian plumage color in a tetrahedral color space: a phylogenetic analysis of new world buntings. *The American Naturalist* 171:755-776.
9. Maia R, Gruson H, Endler JA, & White TE (2019) pavo 2: New tools for the spectral and spatial analysis of colour in r. *Methods in Ecology and Evolution* 10(7):1097-1107.
10. Ödeen A, Håstad O, & Alström P (2011) Evolution of ultraviolet vision in the largest avian radiation - the passerines. *Bmc Evol Biol* 11(1):313.
11. Feng S*, et al.* (2020) Dense sampling of bird diversity increases power of comparative genomics. *Nature* 587(7833):252-257.
12. Revell LJ (2012) phytools: an R package for phylogenetic comparative biology (and other things). *Methods in Ecology and Evolution* 3(2):217-223.
